# Microbial Interactions in Soil

**DOI:** 10.3390/microorganisms10101939

**Published:** 2022-09-29

**Authors:** Volker S. Brözel

**Affiliations:** 1 Departments of Biology and Microbiology, South Dakota State University, Brookings, SD 57006, USA; volker.brozel@sdstate.edu; 2 Department of Biochemistry, Genetics and Microbiology, University of Pretoria, Pretoria 0004, South Africa

Our view on the diversity and distribution of soil microbiota has expanded and continues to do so, driven by high-throughput sequencing technologies, but comparatively little is known about how these organisms affect each other. Bacteria, archaea, fungi, protists and their respective viruses impact each other through a range of beneficial and deleterious interactions, and thereby the soil ecosystem [1]. Modern microbiology, such as agriculture, has been shaped by the mono-culture paradigm, and the secrets of cellular function have been uncovered using a single culture approach. For decades, microbiologists have been trained to obtain and study “pure cultures”, clonal lineages able to grow rapidly on protein-rich laboratory media. In contrast, most microorganisms occur in soil and aquatic environments, surrounded by a myriad of life forms from bacteria, fungi and protists to insects, occurring at high densities amid sparse nutrient availability [2,3]. Bacteria contribute 70 Gt of the 550 Gt of global carbon biomass, together with 7 Gt from Archaea, 12 from fungi and 4 from protists. The terrestrial microbial biomass is estimated to be composed of 7 Gt carbon of bacteria, 0.5 of archaea, 12 of fungi and 1.6 of protists [4], so bacteria constitute the largest part of microbiota, not only by number, but also by biomass. In contrast, humans, the hosts to the most studied microbial ecosystem, make up only 0.06 Gt of carbon.

Food readily available to microbes must be water-soluble and amenable to uptake across membranes, collectively termed low-molecular-weight organic substances (LMWOS). While the bacteria-rich gastrointestinal tract receives nutrient input on a semi-continuous basis, soil receives limited and only periodic nutritional input. Soil contains low concentrations of readily accessible carbon sources such as sugars, carboxylic acids and amino acids, driving competition for nutrients among microbes [5,6]. Microbial populations’ success in soil requires multiple survival skills due to a combination of minimal nutrient input and high cell density. Microbes must have evolved diverse ways to position themselves competitively and find a niche where they can maintain populations over time. The high microbial diversity and population density, combined with a wide range of physicochemical conditions in soil across Earth, suggest a wide array of interactive mechanisms.

Microbiologists have tended to look at species interactions through a competitive or toxin-producing lens, framed perhaps by the search for novel antimicrobial compounds or thoughts that so many different species must become competitive. The rather stable nature of these complex soil microbial communities [7,8,9] suggests that perturbations are not common, at least not among the dominant members. Positive interactions may play a larger role than currently appreciated, but how positive interactions stack up against negative ones is an open question. Progress in this area requires a more comprehensive understanding of the diverse mechanisms underpinning positive and negative microbial interactions in communities and understanding of the details underlying the overall stability of microbial communities.

Intriguing interactive phenomena have been described, and Table 1 attempts to summarize the categories of microbe–microbe interactions reported to date. Yet, we have barely scratched the surface of the array of approaches microbes may employ to attain population success in soils. Such novel interactions may be stumbled upon through the keen observation of cultured isolates, but the Network Analysis of barcoding sequence data such as 16S rRNA and ITS gene pools, as well as shotgun metagenomics, presents a rich source to find and examine possible microbial interactions, both negative and positive [10]. Microbial network analysis, clustering approaches, and community assembly processes [11,12] are examples of approaches from which hypotheses for specific interactions can be identified. Guidelines and resources to analyze ecological networks have recently been offered by Goberna and Verdú [13]. I encourage the microbiology community to take a rigorous look at their favorite soil systems, or to revisit existing datasets, as there are bound to be many as-yet undescribed interactive phenomena.

Both novel and classical approaches to finding and studying microbial interactions of interest will be discussed in this Special Issue regarding microbial interactions in soil. This collection of papers will help to broaden our awareness of the intricate microbial interactions that occur in soil.

## Figures and Tables

**Table 1:** Categories of microbe–microbe interactions reported to date.

Interaction Category	Direct Contact ^1^	Positive	Negative
Gene transfer to recipient			
Conjugation	X	X ^2^	X
Viral infection	X	X	X
Toxin injected into recipient cell			
Type III export system	X		X
Type VI export system	X		X
Contact-dependent inhibition	X		X
Cell–cell adhesins			
Facilitating inter-species association	X	X	
Facilitating intra-species association	X	X	
Electron transport through nanowires	X	X	
Syntrophic interactions			
Obligate syntrophy	X	X	
Non-obligate syntrophy	X	X	
Excreted compounds			
Antibiotics			X
Signals affecting gene expression		X	
Signals affecting motility		X	X
Chelating compounds that sequester elements		X	X
Extracellular enzymes that make nutrients available		X	
Quorum quenching			X
Vesicles facilitating outer membrane exchange		X	X
Endosymbiosis			
Endophytes occurring between eukaryotic cells	X	X	X
Bacteria in eukaryotic cells	X	X	X
Eukaryotes in eukaryotic cells	X	X	X
Bacteria in other bacteria	X	X	X

^1^ Direct cell–cell contact is required for this type of interaction to occur. ^2^ Some interactions in this category have a positive effect, while others have a negative effect.
